# Biomechanical evaluation of a novel individualized zero-profile cage for anterior cervical discectomy and fusion: a finite element analysis

**DOI:** 10.3389/fbioe.2023.1229210

**Published:** 2023-09-07

**Authors:** Yang Wang, Yang Liu, Aobo Zhang, Qing Han, Jianhang Jiao, Hao Chen, Xuqiang Gong, Wangwang Luo, Jing Yue, Xue Zhao, Jincheng Wang, Minfei Wu

**Affiliations:** ^1^ Department of Orthopedics, The Second Hospital of Jilin University, Changchun, China; ^2^ Department of Anesthesiology, The Second Hospital of Jilin University, Changchun, China; ^3^ Department of Endocrinology and Metabolism, First Hospital of Jilin University, Changchun, China

**Keywords:** cervical spine, finite element analysis, anterior cervical discectomy and fusion, Cage, biomechanics

## Abstract

**Introduction:** Anterior cervical discectomy and fusion (ACDF) is a standard procedure for treating symptomatic cervical degenerative disease. The cage and plate constructs (CPCs) are widely employed in ACDF to maintain spinal stability and to provide immediate support. However, several instrument-related complications such as dysphagia, cage subsidence, and adjacent segment degeneration have been reported in the previous literature. This study aimed to design a novel individualized zero-profile (NIZP) cage and evaluate its potential to enhance the biomechanical performance between the instrument and the cervical spine.

**Methods:** The intact finite element models of C3-C7 were constructed and validated. A NIZP cage was designed based on the anatomical parameters of the subject’s C5/6. The ACDF procedure was simulated and the CPCs and NIZP cage were implanted separately. The range of motion (ROM), intradiscal pressure (IDP), and peak von Mises stresses of annulus fibrosus were compared between the two surgical models after ACDF under four motion conditions. Additionally, the biomechanical performance of the CPCs and NIZP cage were evaluated.

**Results:** Compared with the intact model, the ROM of the surgical segment was significantly decreased for both surgical models under four motion conditions. Additionally, there was an increase in IDP and peak von Mises stress of annulus fibrosus in the adjacent segment. The NIZP cage had a more subtle impact on postoperative IDP and peak von Mises stress of annulus fibrosus in adjacent segments compared to CPCs. Meanwhile, the peak von Mises stresses of the NIZP cage were reduced by 90.0–120.0 MPa, and the average von Mises stresses were reduced by 12.61–17.56 MPa under different motion conditions. Regarding the fixation screws, the peak von Mises stresses in the screws of the NIZP cage increased by 10.0–40.0 MPa and the average von Mises stresses increased by 2.37–10.10 MPa.

**Conclusion:** The NIZP cage could effectively reconstruct spinal stability in ACDF procedure by finite element study. Compared with the CPCs, the NIZP cage had better biomechanical performance, with a lower stress distribution on the cage and a more moderate effect on the adjacent segmental discs. Therefore, the NIZP cage could prevent postoperative dysphagia as well as decrease the risk of subsidence and adjacent disc degeneration following ACDF. In addition, this study could serve as a valuable reference for the development of personalized instruments.

## Introduction

Cervical degenerative disease is a chronic, structural deterioration of the cervical spine associated with aging and physiological deterioration (Teraguchi et al., 2014; Theodore, 2020; Lee et al., 2021). MRI screenings have shown that between 47.4% and 86.3% of individuals over the age of 50 have cervical disc degeneration, with the C5/6 segment being the most commonly affected (Teraguchi et al., 2014). The symptoms of cervical degenerative disease typically involve cervical axial pain, numbness and weakness in the limbs, and even neurological deficits, resulting in a significantly decreased quality of life (Theodore, 2020). Conservative treatments are generally effective for patients with mild symptoms or a short duration of the disease; However, surgical intervention is a preferable alternative for patients with cervical degenerative disease suffering from severe neurological symptoms and ineffective conservative management (Scholz et al., 2020; Heijdra Suasnabar et al., 2023).

Anterior cervical discectomy and fusion (ACDF) has been a standard procedure for the treatment of symptomatic cervical degenerative disease (Fraser and Hartl, 2007; Zou et al., 2017; Sun et al., 2018). Cage was first proposed by Bagby et al. and was made of stainless steel with a hollow structure (Bagby, 1988). Since then, the cage has been optimized in terms of materials and processes, which has gradually become the preferred internal fixation device for spinal fusion surgery (Zdeblick and Phillips, 2003; Shen et al., 2022). At present, the most frequently performed internal fixation devices in clinical practice are cage and plate constructs (CPCs). The devices have the capability to directly decompress the nerves, restore the height of intervertebral space, and maintain the mechanical stability of the cervical spine. Nevertheless, previous literature has reported several instrument-related complications, such as dysphagia, cage subsidence, and adjacent segment degeneration (Fountas et al., 2007; Moussa et al., 2018). For conventional CPCs, the contact area between the cage and endplates is limited due to the irregular surface of the upper and lower endplates. This limited contact area, prone to relative stress concentration and uneven distribution, potentially resulting in cage subsidence and instrument fracture (Zhang et al., 2022; Sun et al., 2023).

In recent years, with the refinement of individualized medical models, there has been an increase in the design of individualized spinal instruments (Spetzger et al., 2016). Zhang et al. (2022) constructed a novel individualized titanium mesh that improved the compatibility of the implant with the cervical spine as well as decreased implant-related complications. It has been reported that the titanium plates are an important factor contributing to postoperative dysphagia and heterotopic ossification (Sun et al., 2018; Scholz et al., 2020; Guo et al., 2021). To address this, zero-profile cage has been introduced to replace titanium plate fixation with screw-only fixation, which could prevent complications associated with titanium plates (Sun et al., 2018). However, previous reports have demonstrated a higher risk of subsidence for implantation of zero-profile cage compared to conventional CPCs (Lee et al., 2015; Chen et al., 2016). Therefore, it was necessary to design a novel individualized zero-profile (NIZP) cage to prevent instrument-related complications for ACDF.

Finite element (FE) analysis is a crucial *in vitro* experiment that allows for realistic simulation of spinal surgery and evaluation of the biomechanical performance of the spine. Several studies have investigated the biomechanical effects of internal fixation devices on ACDF using FE analysis (Moussa et al., 2018; Hua et al., 2020; Zhou et al., 2021). Consequently, this study aimed to design a NIZP cage for ACDF and evaluate the biomechanical differences between CPCs and NIZP cage. Additionally, it could provide biomechanical evidence for further optimization of cervical cage.

## Materials and methods

### Finite element model of the cervical spine

A three-dimensional FE model of C3-C7 was first reconstructed based on computed tomography scans with 0.8 mm intervals (Dual Source CT; Siemens, Munich, Germany) of a 32-year-old healthy male volunteer (height: 175 cm; weight: 63 kg). This study was performed in strict accordance with the Declaration of Helsinki (2003) and approved by the Ethics Committee of the Second Hospital of Jilin University (Ethical batch number: SB2020189). All details of the experiment were explained to the volunteer and his informed consent was obtained. The computed tomography data were imported into Mimics software v21.0 (Materialise, Inc., Leuven, Belgium) in DICOM format to reconstruct the geometry of the cervical spine model. The initial C3-C7 model was then smoothed and polished using Magics software v21.0 (Materialise, Inc., Leuven, Belgium). Then, solid models of intervertebral discs, facet joints, and endplates were constructed in 3-Matic software v13.0 (Materialise, Inc., Leuven, Belgium). Afterwards, these components of FE model were meshed in Hypermesh v16.0 (Altair Engineering, Troy, Michigan, United States). The vertebral body, intervertebral disc, facet joints, and endplates were constructed using 3D solid elements with isotropic properties (Hua et al., 2020; Shen et al., 2022; Zhang et al., 2022).

**FIGURE 1:**
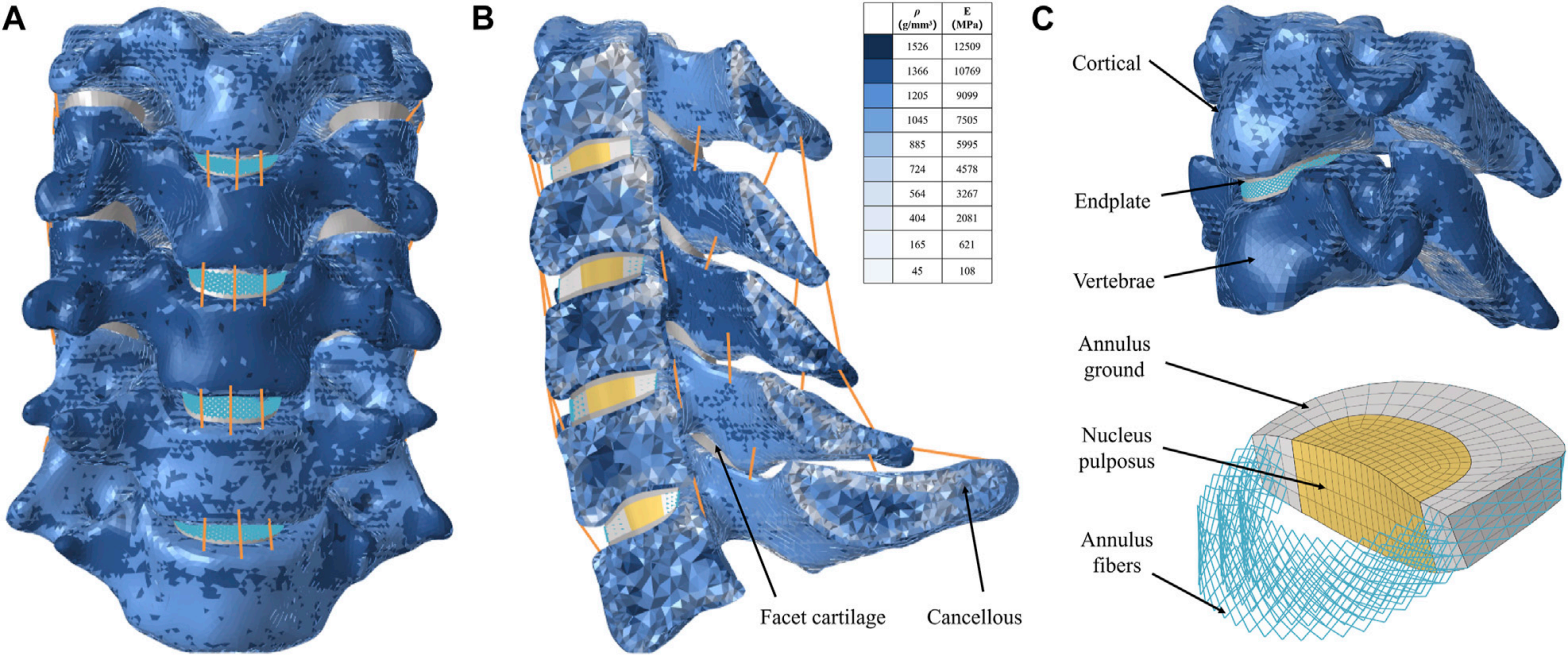
Finite element model for intact C3-7 cervical spine. **(A)** Intact model, **(B)** Left-view section of the finite element model, **(C)** Cortical bone, endplate, annulus ground, nucleus pulposus, and annulus fiber.

The element type of the vertebral body was divided into a four-node tetrahedral mesh (C3D4), and the intervertebral disc, facet joints, and endplates were divided into an eight-node hexahedral mesh (C3D8) (Wo et al., 2021). According to the empirical formulation of Rho et al., the material properties of the vertebrae were attached to the FE model based on computed tomography gray values in Mimics software v21.0 (Rho et al., 1995; Sun et al., 2023). The intervertebral disc was further divided into two parts with a volume ratio of 7:3: nucleus pulposus and annulus fibrosus (Zhang et al., 2022). The nucleus pulposus was modeled as having isotropic, incompressible, fluid-like properties (Kallemeyn et al., 2010). The annulus fibrosus was simulated by annulus fibers wrapped around an annulus fibrosus substance. And the annulus fiber was a mesh structure composed of truss elements that experienced tension only, with an inclination angle between 15° and 45° to the transverse plane (Mo et al., 2017; Zhang et al., 2022). The cervical ligaments, including anterior longitudinal ligament, posterior longitudinal ligament, interspinous ligament, supraspinous ligament, capsular ligament, and ligamentum flavum, were modeled using tension-only truss elements and connected to the adjacent vertebrae (Figure 1) (Shen et al., 2022; Zhang et al., 2022). All components were imported into Abaqus software v6.14 (SIMULIA Inc.) in inp format. The facet joints covered by articular cartilage layer with surface-to-surface contact and a frictional coefficient set at 0.1, and other contact surfaces were defined as Tie contact (Wo et al., 2021; Shen et al., 2022). All material properties and element types of the components of cervical spine were shown in Table 1 (Rho et al., 1995; Wo et al., 2021; Shen et al., 2022; Zhang et al., 2022; Sun et al., 2023).

**TABLE 1:** Material properties of the finite element model.

Component	Element type	Young modulus (MPa)	Poisson’s ratio	Crosssection (mm^2^)	References
**Vertebrae**	C3D4	ρ = 47 + 1.112*HU	0.3	-	Rho et al., 1995; Sun et al., 2023; Wo et al., 2021
		E = 0.63ρ^1.35^			
**Intervertebral disc**					Zhang et al., 2022; Shen et al., 2022
Nucleus pulposus	C3D8	1.0	0.49	-	
Annulus fibers	T3D2	110.0	0.3	-	
Annulus fibrosus substance	C3D8	4.2	0.49	-	
**Endplate**	C3D8	500.0	0.4	-	Wo et al. (2021)
**Facet joint cartilage**	C3D8	10.4	0.4	-	Wo et al. (2021)
**Ligament**					Shen et al., 2022; Zhang et al., 2022
Anterior longitudinal ligament	T3D2	10.0	0.3	6.0	
Posterior longitudinal ligament	T3D2	10.0	0.3	5.0	
Interspinous ligament	T3D2	1.5	0.3	10.0	
Supraspinous ligament	T3D2	1.5	0.3	5.0	
Capsular ligament	T3D2	10.0	0.3	46.0	
ligamentum flavum	T3D2	1.5	0.3	5.0	
**Implants (Ti6Al4V)**	-				Zhang et al. (2022)
NIZP cage, screws	C3D4	110, 000	0.3	-	
CPCs	C3D4	110, 000	0.3	-	

NIZP cage, a novel individualized zero-profile (NIZP) cage; CPCs, cage and plate constructs.

### Design of a novel individualized porous titanium alloy zero-profile cage

The NIZP cage for ACDF was designed in Magics software v21.0 (Materialise, Inc., Leuven, Belgium). Initially, the morphology of the intervertebral space was depicted by extracting the lower surface of the C5 vertebra and the upper surface of the C6 vertebra. Subsequently, a novel cage was designed based on the morphological characteristics of the intervertebral space, aiming to increase the contact area between the implant and the cervical spine. To prevent the cage from entering the spinal canal during fixation screw insertion, an arc-shaped restrictor plate was constructed in front of the cage. The height of the restrictor plate was determined by measuring the intervertebral space height of C5 and C6. Finally, in order to maximize the length of the screw track within the vertebral body and enhance spinal stability, two screws were implanted in the C5 and C6 vertebrae at a 45° angle in the sagittal plane, respectively. The fixation screws had a diameter of 4 mm and a length of 16 mm. Furthermore, the NIZP cage was manufactured from titanium alloy (Ti6Al4V) to enhance the osseointegration performance of the implant (Epasto et al., 2019).

### FE model of ACDF procedure

**FIGURE 2:**
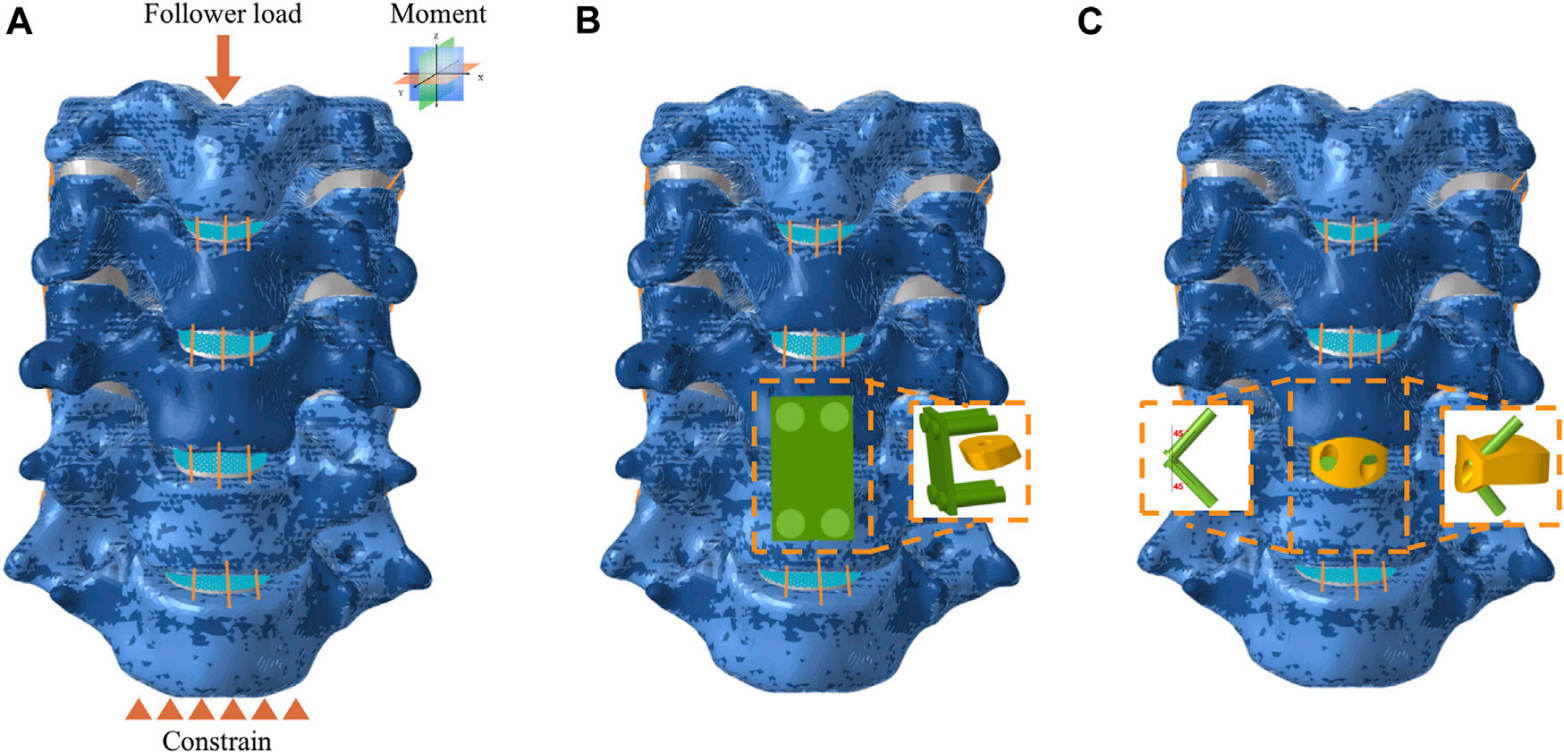
Finite element model for anterior cervical discectomy and fusion. **(A)** Intact model subject to force and constrain, **(B)** CPCs model, **(C)** NIZP cage model.

The ACDF procedure was performed on the C5-C6 segment in the research. At this segment, the anterior longitudinal ligament and intervertebral disc were completely resected. Then two surgical implants, including CPCs and NIZP cage, were simulated and implanted in the C5-C6 intervertebral spaces, respectively (Figures 2B,C). The CPCs are composed of a conventional cage, a titanium plate, and four screws. The titanium plate was fixed anteriorly to the intervertebral space by four screws. As for the NIZP cage, it consists of only a individualized cage and two screws. The cage was implanted into the intervertebral space and secured by two screws. For all surgical models, the contact surfaces between the cage, screws, and the vertebra were defined as Tie contact to simulate complete bony fusion (Zhang et al., 2022). The material properties of these implants are listed in Table 1 (Zhang et al., 2022).

### Mesh convergence

In this research, a mesh convergence test was conducted to validate the influence of mesh refinement on the predictions of the FE model (Shen et al., 2022). The element size of the C3-C7 was set at four different sizes for comparative analysis (Table 2). The element size of the FE model was set at 0.5, 0.8, 1.2, and 1.5 mm in the four cases, respectively. By comparing the peak von Mises stress values predicted by the reference case, the corresponding values of cases A, B, and C were considered accurate within 5% of the reference case. Notably, Case A demonstrated a higher accurate compared to the other cases, maintaining a prediction accuracy of 98% over the reference case model in less computation time.

**TABLE 2:** Mesh convergence test of the mesh density of the FE model.

Case	Element size (mm)	Number of elements	Percentage change in peak von mises stress
Reference case	0.5	211,030	-
Case A	0.8	100,044	<5%
Case B	1.2	50,600	>5%
Case C	1.5	34,754	>5%

### Boundary and loading conditions

As shown in Figure 2A, the intact C3-C7 segment was modeled in the FE analysis. The lower surface of the C7 vertebrae was constrained in all directions, while a follower load of 73.6 N was applied to the upper surface of the C3 vertebra to simulate the weight of the head and muscle force. Moreover, a 1.0 N m moment was performed on the upper surface of the C3 vertebra to simulate flexion, extension, axial rotation, or lateral bending (Sun et al., 2023). The range of motion (ROM) for each segment was calculated based on the relative motions of each vertebra in each motion condition (Panjabi et al., 2001). The ROM of each segment in the intact FE model was compared to previously published data to validate the model’s effectiveness. The differences in biomechanical characteristics of the two surgical implants were compared in each motion condition. Furthermore, the ROM of each segment, intradiscal pressure (IDP) in adjacent segments and peak von Mises stress of the annulus fibrosus in adjacent segments were tested under all motion conditions.

## Results

### Validation of the cervical FE model

**FIGURE 3:**
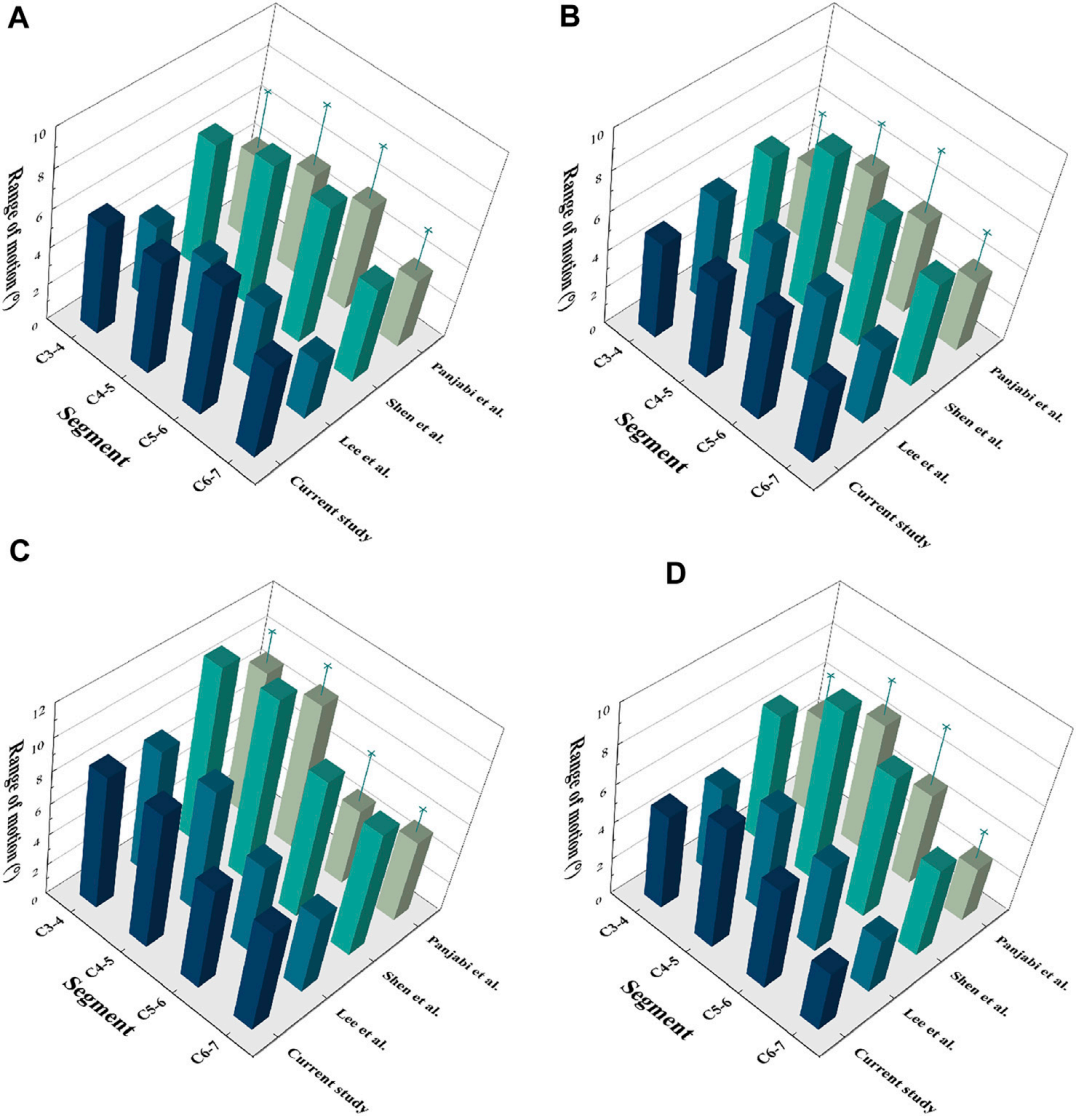
Validation of the C3-7 intact model. **(A)** Flexion, **(B)** Extension, **(C)** Lateral bending, **(D)** Axial rotation.

To validate the cervical FE model, a follower load of 73.6 N and a moment of 1.0 N-m were applied to the upper surface of the C3 vertebrae, while a constraint was applied to the lower surface of C7. The intervertebral ROMs were compared with the results of published *in vitro* experiments as well as FE experiments (Panjabi et al., 2001; Lee et al., 2016; Shen et al., 2022) (Figure 3). The ROMs of the intact model at C3/4, C4/5, C5/6, and C6/7 were 5.85°, 6.09°, 7.12°, and 5.20°, respectively, in flexion; 5.06°, 5.35°, 5.68°, and 4.21°, respectively, in extension; 8.52°, 8.72°, 5.62°, and 4.76°, respectively, in lateral bending; and 5.06°, 6.58°, 5.54°, and 3.34°, respectively, in axial rotation. The prediction results of the cervical FE model were consistent with the results reported in the previous literature.

### ROMs after surgery

**FIGURE 4:**
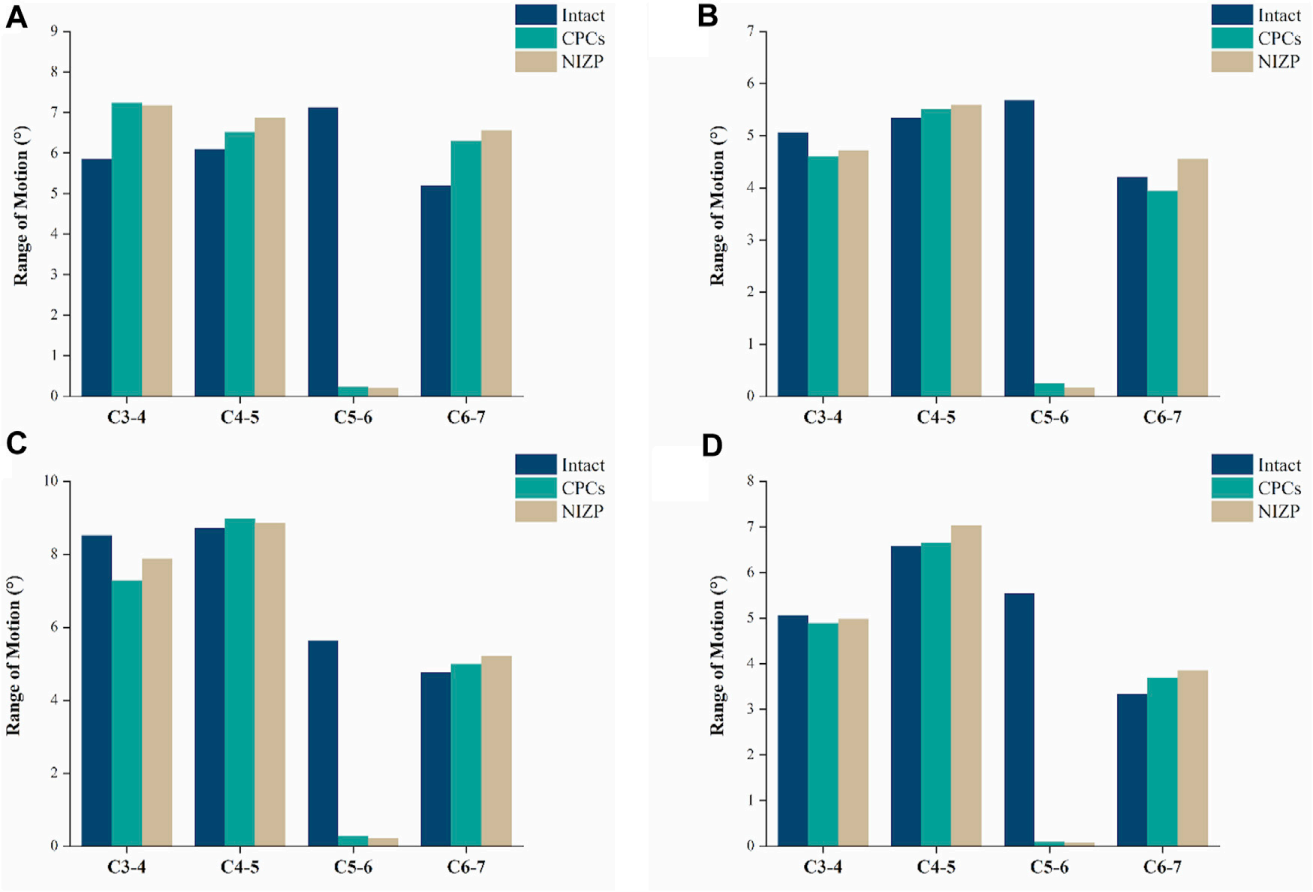
Comparison of the range of motion for the three models under four motion conditions. **(A)** Flexion, **(B)** Extension, **(C)** Lateral bending, **(D)** Axial rotation.

As shown in Figure 4, the ROMs at C5/6 for the intact, CPCs and NIZP cage models were 7.12°, 0.24°, and 0.20° in flexion; 5.68°, 0.25°, and 0.16° in extension; 5.62°, 0.28°, and 0.22° in lateral bending; and 5.54°, 0.10°, and 0.08° in axial rotation, respectively. Compared to the intact model, the ROMs of the two surgical models were significantly decreased under four motion conditions. In addition, postoperative ROMs in adjacent segments increased in both the CPCs and NIZP groups, especially in flexion.

### Intradiscal pressure in adjacent segments

**FIGURE 5:**
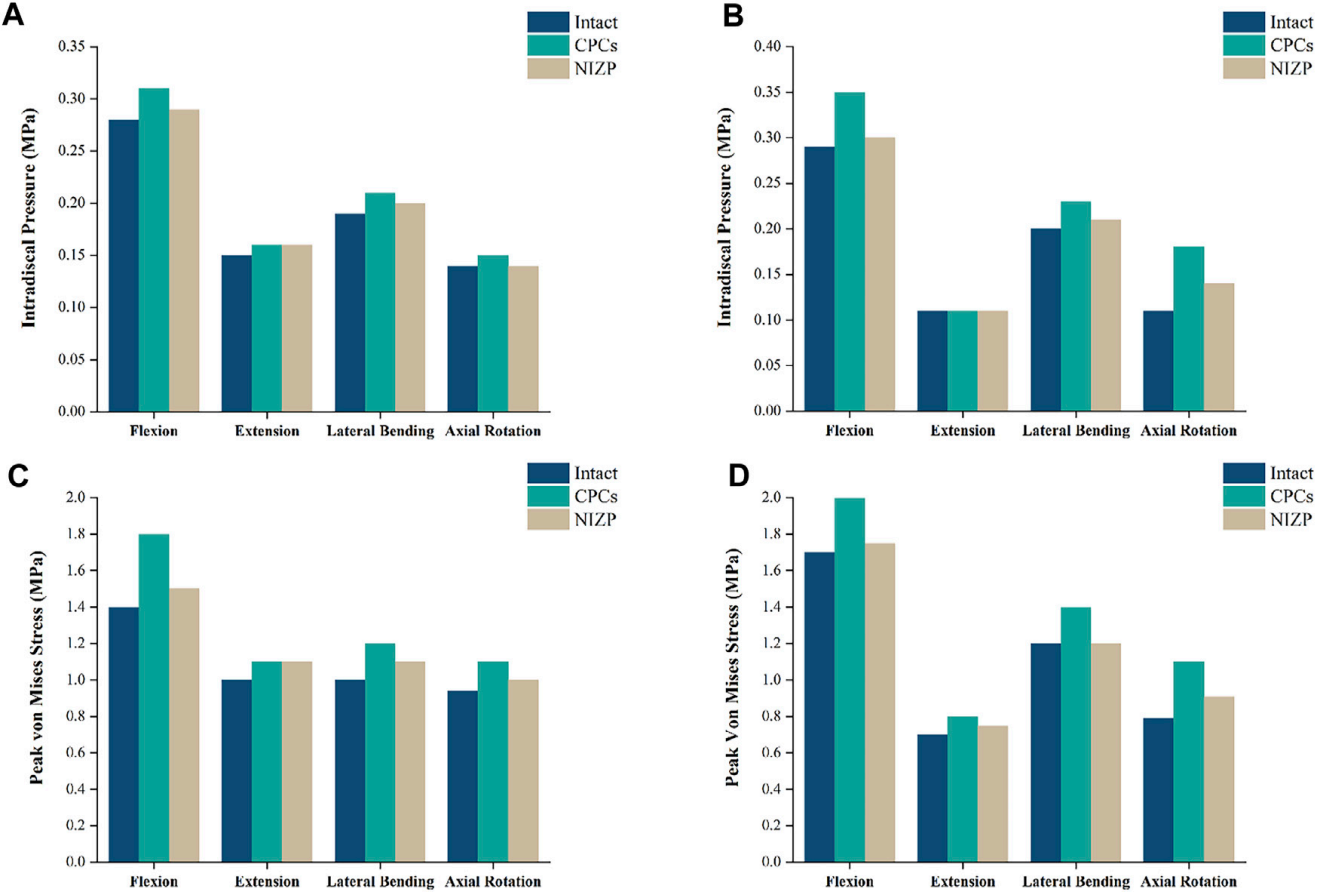
Comparison of intradiscal pressure and peak von Mises stresses in adjacent segments for the three models under four motion conditions. **(A)** IDP in C4/5, **(B)** IDP in C6/7, **(C)** Peak von Mises stresses in C4/5, **(D)** Peak von Mises stresses in C6/7.

**FIGURE 6:**
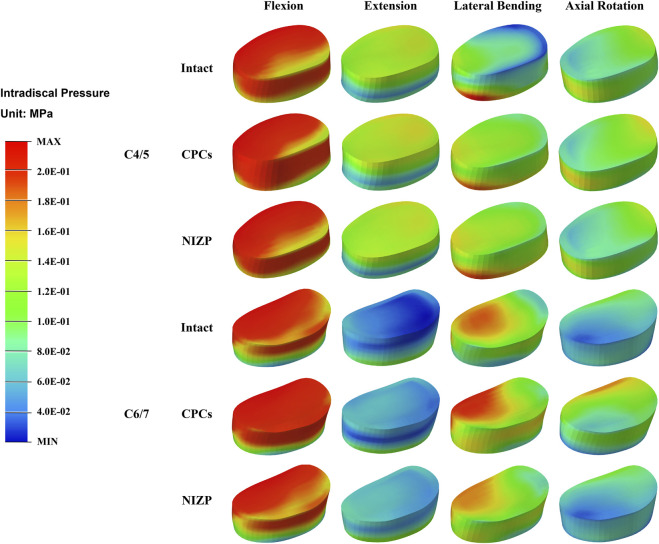
The stress distribution of disc in adjacent segments for the three models under four motion conditions.

IDP at C4/5 and C6/7 are presented in Figures 5A,B. At C4/5, the IDP of the intact, CPCs and NIZP cage models were 0.28 MPa, 0.31 MPa, and 0.29 MPa in flexion; 0.15 MPa, 0.16 MPa, and 0.16 MPa in extension; 0.19 MPa, 0.21 MPa, and 0.20 MPa in lateral bending; and 0.14 MPa, 0.15 MPa, and 0.14 MPa in axial rotation, respectively. As for C6/7, the IDP of three models were 0.29 MPa, 0.35 MPa, and 0.30 MPa in flexion; 0.11 MPa, 0.11 MPa, and 0.11 MPa in extension; 0.20 MPa, 0.23 MPa, and 0.21 MPa in lateral bending; and 0.11 MPa, 0.18 MPa, and 0.14 MPa in axial rotation, respectively. Compared with the intact model, the IDP in adjacent segments increased in both surgical models under four motion conditions, with a more pronounced variation in the CPCs mode (Figure 6).

### Peak von mises stress of annulus fibrosus in adjacent segments

**FIGURE 7:**
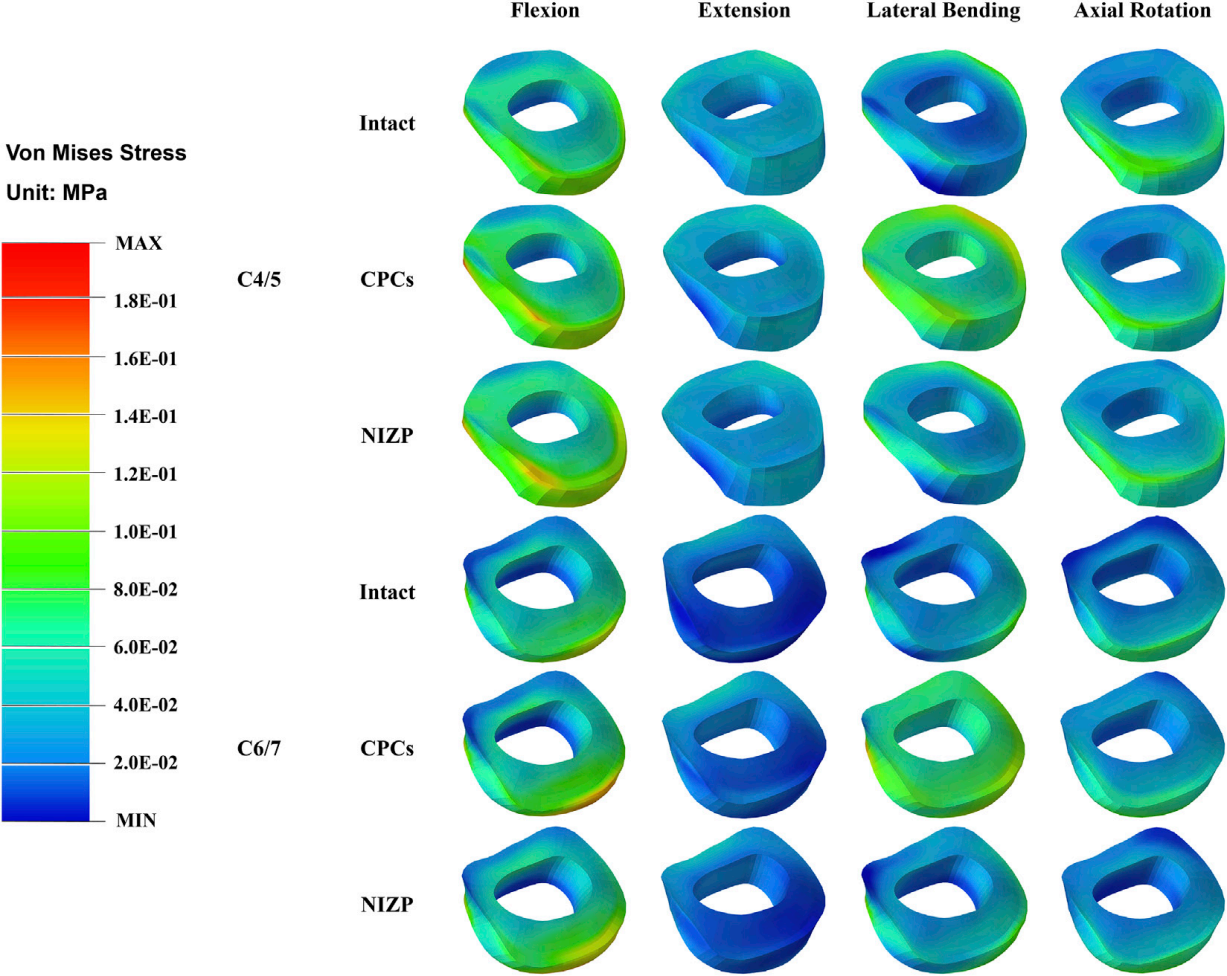
The stress distribution of annulus fibrosus in adjacent segments for the three models under four motion conditions.

Peak von Mises stress on the annulus fibrosus at C4/5 and C6/7 are presented in Figures 5C,D. For C4/5, the peak von Mises stresses of annulus fibrosus of the intact, CPCs and NIZP models were 1.40 MPa, 1.80 MPa, and 1.50 MPa in flexion; 1.00 MPa, 1.10 MPa, and 1.10 MPa in extension; 1.00 MPa, 1.20 MPa, and 1.10 MPa in lateral bending; and 0.94 MPa, 1.10 MPa, and 1.05 MPa in axial rotation, respectively. At C6/7, the peak stresses of annulus fibrosus of three models were 1.70 MPa, 2.00 MPa, and 1.75 MPa in flexion; 0.70 MPa, 0.80 MPa, and 0.75 MPa in extension; 1.20 MPa, 1.40 MPa, and 1.20 MPa in lateral bending; and 0.79 MPa, 1.10 MPa, and 0.91 MPa in axial rotation, respectively. Peak von Mises stresses on the annulus fibrosus of adjacent segments were increased owing to internal fixation devices, especially the CPCs. The stress cloud maps of the annulus fibrosus are shown in Figure 7.

### Von mises stress of internal fixation systems

**FIGURE 8:**
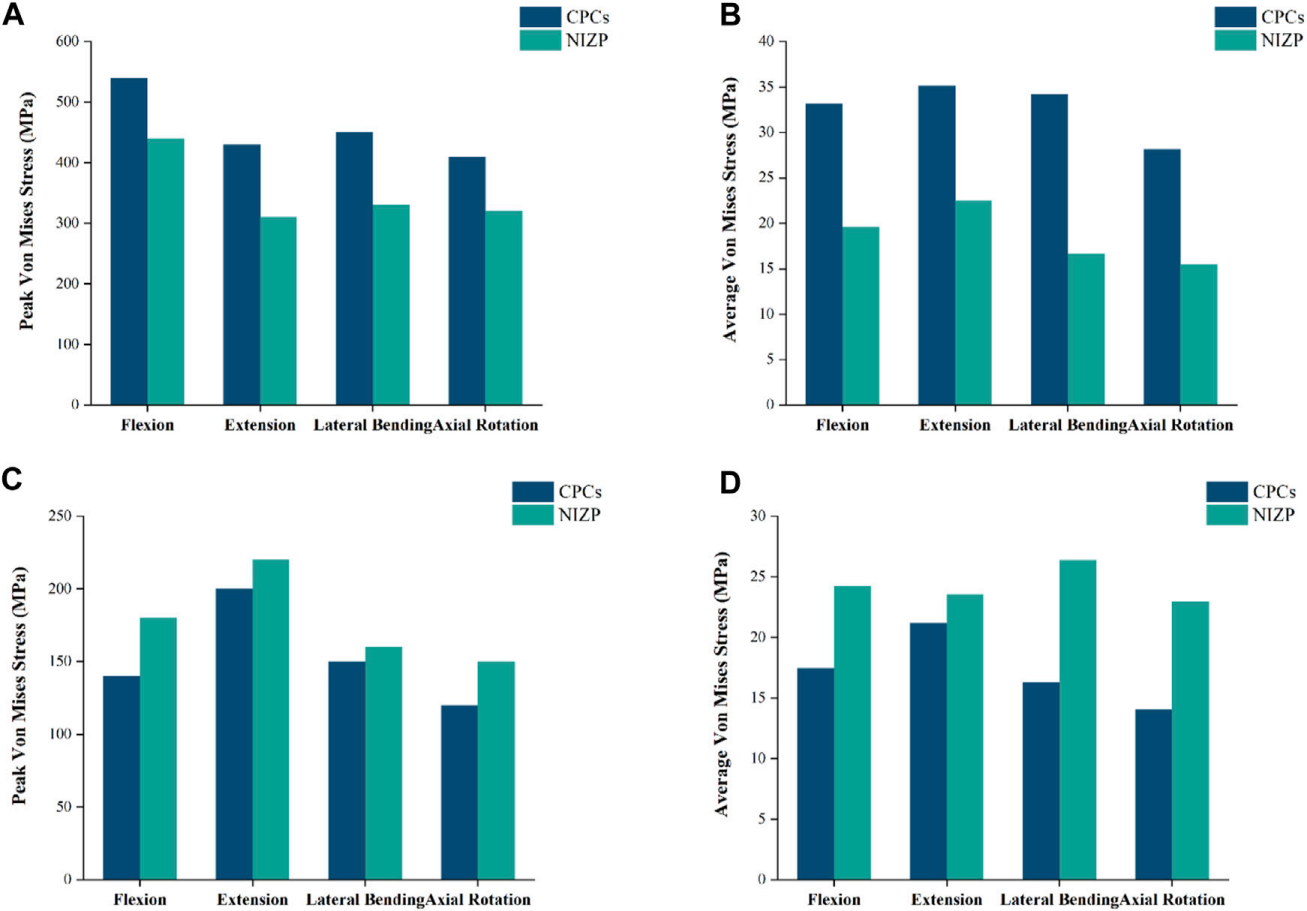
Comparison of peak or average von Mises stresses for the CPCs and NIZP cage under four motion conditions. **(A)** Peak von Mises stresses for cage of the two instrument, **(B)** Average von Mises stresses for cage of the two instrument, **(C)** Peak von Mises stresses for fixation screws of the two instrument, **(D)** Average von Mises stresses for fixation screws of the two instrument.

**FIGURE 9:**
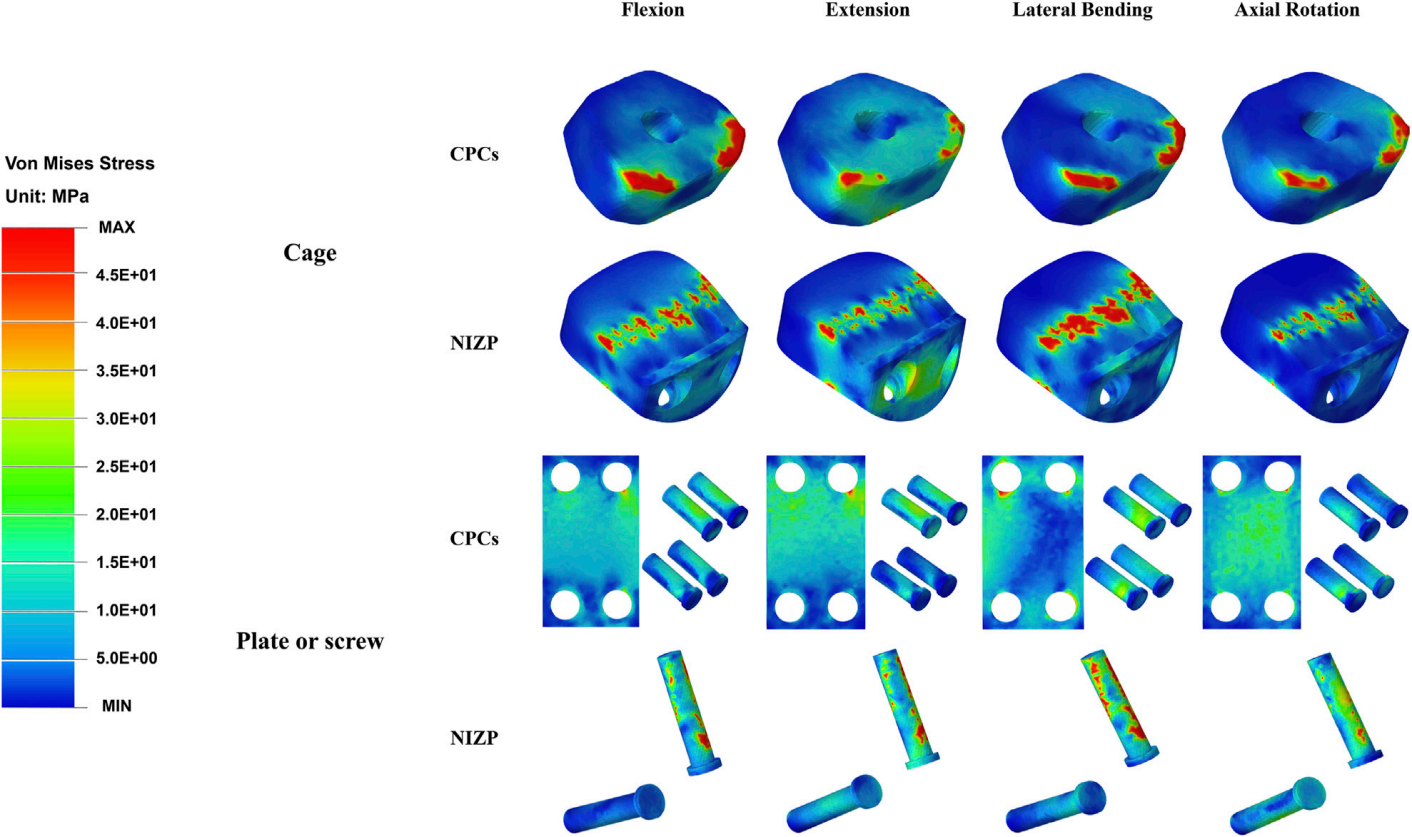
The stress distribution of the CPCs and NIZP cage under four motion conditions.

As shown in Figure 8, the peak and average von Mises stresses of two internal fixation systems - CPCs and NIZP - are compared under different motion conditions. Among them, the peak von Mises stresses in the cage for the CPCs and NIZP models were 540.0 MPa and 440.0 MPa, 430.0 MPa and 310.0 MPa, 450.0 MPa and 330.0 MPa, and 410.0 MPa and 320.0 MPa in flexion, and extension, lateral bending, and axial rotation, respectively. In addition, the average von Mises stresses in the cage for two surgical models were 33.17 MPa and 19.61 MPa, 35.10 MPa and 22.49 MPa, 34.19 MPa and 16.63 MPa, and 28.15 MPa and 15.48 MPa under four motion conditions, respectively. As for plate or screw, the peak von Mises stresses for two surgical models were 140.0 and 180.0 MPa, 200.0 and 220.0 MPa, 150.0 and 160.0 MPa, and 120.0 and 150.0 MPa under four motion conditions, respectively. And the average von Mises stresses in cage for two surgical models were 17.46 MPa and 24.24 MPa, 21.20 MPa and 23.57 MPa, 16.28 MPa and 26.38 MPa, and 14.07 MPa and 22.97 MPa under four motion conditions, respectively. The stress distributions of the internal fixation systems are shown in Figure 9.

## Discussion

Anterior cervical discectomy and fusion (ACDF) is widely acknowledged as the most common and effective treatment for cervical degenerative diseases (Zou et al., 2017; Sun et al., 2018). CPCs plays an important role in ACDF. Previous studies have demonstrated that the implantation of titanium plates in the anterior approach stabilizes spinal structures and promotes fusion (Zou et al., 2017; Guo et al., 2021). However, this approach may irritate the esophagus and increase the risk of postoperative dysphagia (Xiao et al., 2017; Liu et al., 2020). To address these concerns, the NIZP cage was designed to decrease implant-related complications. In this study, a finite element model of C3-C7 segment was constructed to evaluate the biomechanical performance of the NIZP cage. In ACDF procedure, surgeons only need to expose the prevertebral soft tissue of diseased segment to implant the NIZP cage, thereby reducing excessive irritation of the esophagus and minimizing postoperative dysphagia. ROM was evaluated in this study to determine the efficacy of implants in maintaining structural stability of the cervical spine. The results revealed that both the implants effectively reduced the ROM at the surgical segment under four motion conditions, compare to the intact model. Moreover, the ROMs of the NIZP cage was slightly lower than that of the CPCs. It was relevant to a better fit with the upper and lower cervical endplates. However, there was no statistically significant difference between the two models. Zhang et al. (2016) conducted FE analysis and cadaveric studies to demonstrate that a cage that closely matched the cervical spine provided better stability during flexion and extension motion compared to the conventional cage. It is worth mentioning that the ROMs at the C4/5 and C6/7 were increased in both surgical models, especially in flexion and extension motion. To compensate for the lost ROM in the surgical segment, the cervical spine increased the ROM in the adjacent segment to maintain postoperative ROM. Previous *in vitro* mechanical experiments (Eck et al., 2002) and FE analysis (Zhou et al., 2021) similarly concluded that there was a corresponding increase in the mobility of the upper and lower segments after ACDF. In general, the stability of spinal structure was well reconstructed by both CPCs and NIZP cage after ACDF.

Stress distribution is frequently utilized in FE studies to evaluate the risk of subsidence and fixation failure, with von Mises stress serving as a crucial indicator (Wo et al., 2021). Following ACDF, cage subsidence is a prevalent complication, with reported subsidence rates ranging from 8% to 34%. This subsidence could contribute to kyphotic deformity, nerve impairment, *etc.* (Zhang et al., 2016; Sun et al., 2018; Jin et al., 2021; Zhang et al., 2022). Inadequate compatibility between the cage and the spine endplate leads to uneven stress distribution and stress concentration, ultimately posing a potential risk of cage subsidence (Shen et al., 2022; Zhang et al., 2022). A NIZP cage was constructed in this study based on the anatomical characteristics of the cervical spine, in order to enhance the compatibility between the cage and the endplate. Comparing the stress distributions of the two implants, it revealed that the NIZP cage had a reduction in peak von Mises stresses by 90.0–120.0 MPa and average von Mises stresses by 12.61–17.56 MPa compared to CPCs under the four motion conditions. As shown in the stress cloud maps, the stresses in CPCs were mainly concentrated in the edge areas, particularly in the front area of the cage. In contrast, the stresses in the NIZP cage were more evenly distributed in the inner areas and the anterior restrictor plate. This indicated that a well-matched implant could decrease the peak stress and improve stress distribution, thereby reducing the risk of cage subsidence. Several studies also support this perspective (Zhang et al., 2022; Sun et al., 2023), stating that increasing the contact area through improved conformity of the implant to the endplate can prevent excessive stress concentration and reduce the risk of implant subsidence.

Disc degeneration in adjacent segments represents an important complication after spinal surgery (Fountas et al., 2007). To assess the risk of adjacent segment degeneration, the IDP and the peak von Mises stresses of annulus fibrosus in the adjacent segments were measured respectively in this study. For both surgical groups, the postoperative IDPs of C4/5 and C6/7 were higher than those of the intact model. And the IDP of the CPCs group were slightly higher than those of the NIZP group under most motion conditions. Specifically, under flexion and axial rotation conditions, the IDP in the CPCs group increased by 7.14% in C4/5 segment, and by 17.24% and 36.36% in C6/7 segment, respectively, compared to the NIZP group. It is well known that the motion unit of the cervical spine consists of the upper and lower vertebrae and an intervertebral disc (Theodore, 2020). After ACDF, an intervertebral disc structure was sacrificed and replaced by a titanium alloy. The loss of a motion unit led to a corresponding increase in ROMs of adjacent segments, which resulted in an increase in IDP and annulus fibrosus stresses (Eck et al., 2002). Similarly, Zhang et al. (2022) concluded that the increase in ROM of the adjacent segments after surgery contributed to further disc compression or stretching, resulting in increased stresses. Overall, the NIZP cage facilitates the decrease of the risk of disc degeneration in the adjacent segment after ACDF compared to CPCs.

In addition, fixation screws are an important component of the two implants. Compared to the screw-plate device of CPCs, the NIZP cage exhibited an increase in peak von Mises stresses in the screws within a range of 10.0–40.0 MPa, and an increase in average von Mises stresses within a range of 2.37–10.10 MPa under various motion conditions. As shown in the stress cloud map, the stresses of the screw-plate device were primarily concentrated in the contact area between the screws and the titanium plate under different motion conditions. However, in the contact area between the screws and the vertebral body, the stresses were distributed more uniformly. On the contrary, the stresses in the screws of the NIZP cage were primarily concentrated in the contact area between the screws and the vertebral body, especially in the upper screws. Since the fixation pattern of NIZP cage relied on only two screws, it was inevitable that the stress increased and the stress concentrated in the screws. It is worth mentioning that the peak von Mises stress in the screw is far less than the yield strength of the titanium alloy, which is not sufficient to cause screw fracture or failure (Zhang et al., 2018).

The study has several limitations that should be acknowledged. Firstly, only FE analysis was performed to test the biomechanical performance of the NIZP cage. However, further validation is needed through animal and clinical experiments. Secondly, the finite element model was simplified within an acceptable range, including material properties, boundary conditions, and contact relations. The influence of the cervical muscles on the biomechanics was not considered, which means that the study cannot completely simulate the actual situation after ACDF. Thirdly, although the cages used in the study have porous structures, solid structures were employed for all the analyses to ensure better convergence of the calculations. And the fourth, the finite element analysis was based on data from only one patient. Additionally, *in vitro* biomechanical experiments and clinical studies will be conducted in the future to evaluate the findings of this study.

## Conclusion

The NIZP cage could effectively reconstruct spinal stability after ACDF by FE analysis. The NIZP cage demonstrated superior biomechanical performance compared to CPCs, resulting in a lower stress distribution on the cage and a more moderate effect on the adjacent segmental discs. Therefore, the NIZP cage could prevent postoperative dysphagia as well as decrease the risk of subsidence and adjacent disc degeneration after ACDF. In addition, this study could serve as a valuable reference for the development of personalized instruments.

## Data Availability

The original contributions presented in the study are included in the article/Supplementary Material, further inquiries can be directed to the corresponding authors.
